# Resting heart rate: its correlations and potential for screening metabolic dysfunctions in adolescents

**DOI:** 10.1186/1471-2431-13-48

**Published:** 2013-04-05

**Authors:** Rômulo A Fernandes, Enio R Vaz Ronque, Danielle Venturini, Décio S Barbosa, Danilo P Silva, Crisieli T Cogo, Mariana Souza Carnelossi, Mariana B Batista, Manuel J Coelho-e-Silva, Luís B Sardinha, Edilson S Cyrino

**Affiliations:** 1 Scientific Research Group Related to Physical Activity (GICRAF). Laboratory of Investigation in Exercise (LIVE). Department of Physical Education, Center of Sciences and Technology, UNESP Univ Estadual Paulista, Presidente Prudente, Brazil; 2 Study and Research Group in Metabolism, Nutrition, and Exercise – GEPEMENE. State University of Londrina – UEL, Londrina, Brazil; 3 Department of Physical Education, Center of Physical Education and Sport, State University of Londrina – UEL, Londrina, Brazil; 4 Department of Pathology, Clinical and Toxicological Analysis. Center of Health Sciences, University Hospital, State University of Londrina – UEL, Londrina, Brazil; 5 Faculty of Sport Sciences and Physical Education. University of Coimbra, Coimbra, Portugal; 6 Exercise and Health Laboratory, Faculty of Human Kinetics, Technical University of Lisbon, Lisbon, Portugal

**Keywords:** Heart rate, Adipose tissue, Sleep, Physical fitness, Lipid, Glucose, Adolescent

## Abstract

**Background:**

In pediatric populations, the use of resting heart rate as a health index remains unclear, mainly in epidemiological settings. The aims of this study were to analyze the impact of resting heart rate on screening dyslipidemia and high blood glucose and also to identify its significance in pediatric populations.

**Methods:**

The sample was composed of 971 randomly selected adolescents aged 11 to 17 years (410 boys and 561 girls). Resting heart rate was measured with oscillometric devices using two types of cuffs according to the arm circumference. Biochemical parameters triglycerides, total cholesterol, high-density lipoprotein cholesterol, low-density lipoprotein cholesterol and glucose were measured. Body fatness, sleep, smoking, alcohol consumption and cardiorespiratory fitness were analyzed.

**Results:**

Resting heart rate was positively related to higher sleep quality (β = 0.005, *p* = 0.039) and negatively related to cardiorespiratory fitness (β = −0.207, *p* = 0.001). The receiver operating characteristic curve indicated significant potential for resting heart rate in the screening of adolescents at increased values of fasting glucose (area under curve = 0.611 ± 0.039 [0.534 – 0.688]) and triglycerides (area under curve = 0.618 ± 0.044 [0.531 – 0.705]).

**Conclusion:**

High resting heart rate constitutes a significant and independent risk related to dyslipidemia and high blood glucose in pediatric populations. Sleep and cardiorespiratory fitness are two important determinants of the resting heart rate.

## Background

Early life is a determinant period in the prevention [1,2] and development [3] of chronic diseases in adulthood and, therefore, the development of inexpensive tools to identify youth at an increased risk are useful in epidemiological and clinical settings. As a result of this point of view, anthropometric variables have been tested and widely used for screening those with an increased cardiovascular risk [4-6].

More recently, resting heart rate (RHR) has been suggested as a valuable indicator of risk. Among adults there is scientific evidence to suggest that tachycardia should no longer be viewed as an innocent clinical feature [7]. Similarly, increased values of RHR constitute a significant risk factor in the development of cardiovascular outcomes, such as heart failure, myocardial infarction, sudden cardiac death and stroke (independent of blood pressure and a variety of other risk factors) [8]. However, the literature on this topic is relatively limited in pediatric populations.

A previous study [9] found a significant relationship between high RHR and elevated blood pressure in 356 male children and adolescents. Surprisingly, the authors observed that this association occurred in both obese and lean boys. Rabbia et al. [10] also found a positive association between RHR and elevated blood pressure in adolescents of both sexes. Similarly, there is a positive relationship between RHR and lipid variables in obese children and adolescents [11].

The above mentioned data is in favor of the use of RHR as an index in screening pediatric populations at an increased risk. However, since RHR has not been thoroughly studied in epidemiological studies, the determinants of RHR in pediatric populations need to be further clarified. Therefore, the purposes of this study were to analyze the impact of resting heart rate for screening dyslipidemia and high blood glucose and also to identify its significance in pediatric populations.

## Methods

### Sample

This was a school based study, in which the sample was composed of adolescents (11 – 17 years-old) of both genders from Londrina, Brazil; which is a medium-sized city (~500,000 inhabitants) located in South Brazil with a high human development index (0.824) [12]. The minimum sample size of 554 adolescents was estimated using an equation for correlation coefficients, adopting *r* = 0.18 [11], power of 80% and an alpha error of 5% (sample size was increased by 100% due to design effect and by 30% for predictable losses).

The sample of schoolchildren was selected in 2011, through a sampling process involving two random stages. The city was divided into five geographical regions (east, west, north, south and center) and two or three schools in each geographical region were randomly selected to participate in the survey. In each of the selected schools, individual classes were randomly selected and thereafter all students in the chosen classes were invited to participate. The inclusion criteria were: (i) self-report of health (absence of previously detected chronic diseases: high blood pressure, diabetes mellitus, any type of dyslipidemia or asthma); (ii) aged between 11–17 years-old. Initially, 1,396 adolescents of both genders agreed to participate and returned the completed, signed consent form. However, 425 boys and girls were later excluded (e.g. absence in the fasting blood sample measurement; lack of 10–12 hours of fasting; refusal to participate in the running test). Therefore, after the field work, 971 adolescents (Male: 42.2% [n = 410] and Female: 57.8% [n = 561]) composed the sample.

A comprehensive verbal description of the nature and purpose of the study, as well as the clinical implications of the investigation, was provided to the participants, their parents and teachers. Written informed consent was obtained from the adolescent’s parent or legal guardian and all participants gave verbal consent. This study was approved by the local ethical committees and all procedures were in accordance with those outlined by the Declaration of Helsinki.

### Independent variables

In this study, six independent variables were taken into account: body fatness percentage (%BF), sleep pattern, sport practice, cardiorespiratory fitness, cigarette and alcohol consumption.%BF was estimated using an equation based on skinfold thickness specifically for children and adolescents [13]. Sleep pattern was assessed by the question “Do you have trouble sleeping?”, with responses based on the likert scale (never [score 1], sometimes [score 2], very often [score 3] and always [score 4]). Sport practice was assessed by the score from section 2 of the Baecke questionnaire [14] and cardiorespiratory fitness was estimated by a maximal multistage 20-meter shuttle run test, in which the peak oxygen uptake (in mL/kg/min) was estimated using a specific equation [15,16]. The number of cigarettes and alcoholic drinks consumed in the previous week was computed.

### Resting heart rate

Oscillometric devices (Omron MX3 Plus), clinically validated for measuring blood pressure in adolescents [17], were used to measure RHR (expressed as beats per minute [beats/min]) and two types of cuffs were used according to the arm circumference (6 mm × 12 mm and 9 mm × 18 mm). To determine which cuff would be used, the circumference of the arm of each child was measured, and the cuff that was approximately 40% of the width of the arm circumference and 80% of the length was used [17]. All measurements were registered in a quiet room with the adolescents resting in the sitting position for 5 minutes with their back supported and feet on the ground. Two measures were taken and the mean value of both was utilized.

There are not any widely accepted RHR cutoffs, therefore, RHR values were stratified into quartiles provided by a previous study [9]: <70 beats/min; 70–77.4 beats/min; 77.5-85.9 beats/min; ≥86 beats/min. The above mentioned quartiles were adopted because both (i) were generated in a dataset which constituted Brazilian children and adolescents and (ii) have been associated with high blood pressure independent of obesity status.

### Blood samples

After fasting for 10–12 hours, the adolescents’ blood samples were collected in tubes containing ethylene-diamine-tetraacetic acid (EDTA) as an anticoagulant and antioxidant, kept on melting ice during transfer, and immediately processed to obtain plasma, using a refrigerated centrifuge 4°C (Fanem®), and stored at −80°C (Indrel®) until the assay was performed. All collected blood samples (performed by nurses) were performed at the patients’ school and biochemical analyses were done at the University Hospital at the Center of Health Sciences at the Universidade Estadual de Londrina. Biochemical parameters, including serum triglycerides, total cholesterol (TC), high-density lipoprotein cholesterol (HDL-C), low-density lipoprotein cholesterol (LDL-C), and glucose were measured by a biochemical autoanalyser (Dimension®, RXL, Newark, NJ, USA) and were used in conjunction with Dade Behring – Siemens kits. Modifications in lipid profile (TC ≥170 mg/dL, LDL ≥130 mg/dL, HDL < 45 mg/dL and triglycerides ≥130 mg/dL) and fasting glucose (≥100 mg/dL) were identified [18].

### Potential confounders

Chronological age, pubertal stage, gender and cardiorespiratory fitness were used as potential confounders and, therefore, adjusted for in the multivariable models. Chronological age was determined as a decimal variable using the difference between the birthday and the date of the assessment. Pubertal stage was identified by the peak height velocity, which was used to estimate biological maturity. The technique estimates time before or after the peak height velocity from the chronological age and anthropometric measures (height, sitting height, estimation of leg length and body weight) as described by Mirwald et al. [19].

### Statistical procedures

The Kolmogorov-Smirnov test analyzed the distribution of the numerical variables and, when necessary, logarithm transformation was used on variables with non-parametric distribution. Analysis of variance using Tukey’s post hoc test compared numerical variables. Pearson correlation assessed the relationship between numerical variables and a linear regression model was elaborated with variables statistically significant in the Pearson correlation (RHR treated as dependent variable). The Chi-square test assessed association among categorical variables and the binary logistic regression (odds ratio [OR] and its 95% confidence interval [OR_95%CI_]) indicated the magnitude of these associations (RHR treated as an independent variable). Gender, age and pubertal stage adjusted both multivariable models (linear regression and binary logistic regression). Additionally, binary logistic regression was adjusted by cardiorespiratory fitness. The receiver-operating characteristic (ROC) curve (expressed as the area under the ROC curve [AUC]) analyzed the potential of RHR for screening metabolic outcomes. Statistical significance was set at *p* < .05 and statistical software BioEstat version 5.0 (BioEstat, Tefé, Amazonas) was used for all analyses.

## Results

The sample in this study was composed of 971 adolescents aged 11 to 17 years (410 boys and 561 girls). The mean age and mean RHR were 12.9 ± 1.4 years-old and 82.7 ± 12.5 beats/min, respectively. The general characteristics of the adolescents stratified by RHR values are presented in Table 1.

**Table 1:** General characteristics of the adolescents stratified by resting heart rate values (Brazil, n = 971)

**Independent variables**	**<70 beats/min Mean (SD)**	**70-77.4 beats/min Mean (SD)**	**77.5-85.9 beats/min Mean (SD)**	**≥86 beats/min Mean (SD)**	***F***	***p***
Age (years)	13.6 (1.6)^a^	13.1 (1.4)^a^	13.0 (1.4)^a^	12.6 (1.2)	20.057	0.001
%BF	20.5 (7.1)^a^	22.1 (7.1)	23 (7.4)	23.1 (7.5)	5.214	0.001
VO_2peak_ (mL/kg/min)	40.5 (5.1)^a^	39.8 (5.3)	39.3 (4.9)	39.1 (4.2)	2.992	0.030
LDL-C (mg/dL)	95.5 (23)	97.4 (24)	99.1 (24)	100.8 (25)	1.886	0.130
HDL-C (mg/dL)	52.2 (12.9)	52.1 (12.7)	51.5 (13.5)	51.8 (13.1)	0.119	0.949
Triglycerides (mg/dL)	58.8 (26.8)^a^	60.5 (26.7)	65.5 (36.2)	69.5 (53.3)	3.348	0.019
TC (mg/dL)	159.5 (27.2)^a^	161.6 (27.4)	163.6 (27.6)	166.7 (27.7)	2.841	0.037
Glucose (mg/dL)	88.2 (6.3)	89.7 (16.1)	90.6 (15.7)	90.1 (6.4)	1.320	0.267

SD = standard deviation; LDL-C = low density lipoprotein cholesterol; HDL-C = high density lipoprotein cholesterol; TC = total cholesterol;%BF = percentage of body fatness; VO_2_peak = peak oxygen uptake; F = one way ANOVA; ^a^ = Tukey’s post hoc test with *p*-value <0.05 compared to the group ≥86 beats/min.

RHR was positively and significantly related to%BF and sleep disorders. Sport practice and cardiorespiratory fitness were positively related (*r* = 0.18; *p* = 0,001). Similarly, RHR was negatively and significantly related to cardiorespiratory fitness, sport practice and alcohol consumption. The number of cigarettes was not related to RHR values. Age (*r* = −0.24; *p* = 0.001) and pubertal stage (*r* = −0.09; *p* = 0.002) was negatively related to RHR, on the other hand, male gender (*r* = 0.14; *p* = 0.001) was significantly and positively related to RHR. In the multivariable model, independent of the other variables, only cardiorespiratory fitness and sleep disorder remained significantly related to RHR (Tables 2 and 3).

**Table 2:** Relationship between resting heart rate and independent variables among adolescents (Brazil, n = 971)

**Independent variables**	**Correlation coefficient**		**Linear regression**	
	***r***	***p***	**β**_adjusted_*	***p***
%BF	0.10	0.001	0.003	0.845
VO_2peak_	−0.09	0.004	−0.207	0.001
Sport practice	−0.08	0.008	−0.006	0.295
Alcohol	−0.08	0.014	−0.001	0.225
Cigarettes	−0.04	0.162	---	--
Sleep pattern	0.12	0.001	0.005	0.039

*Multivariable model in which all independent variables were entered simultaneously, in addition to being adjusted by age, pubertal stage and gender.%BF = percentage of body fatness; VO_2_peak = peak oxygen uptake; Alcohol = number of drinks per week; Cigarettes = number of cigarettes per week. Statistical significance was set at *p* < 5%.

**Table 3:** Relationship between resting heart rate and metabolic variables among adolescents (Brazil, n = 971)

**Independent variables**	**Correlation’s coefficient**		**Linear regression**	
	***r***	***P***	**β**_adjusted_*	***p***
LDL-C	0.05	0.131	---	---
HDL-C	−0.01	0.783	---	---
Triglycerides	0.08	0.011	0.019	0.049
TC	0.08	0.007	0.017	0.252
Glucose	0.07	0.017	0.031	0.341

*Multivariable model adjusted by age, pubertal stage, gender and cardiorespiratory fitness; LDL-C = low density lipoprotein cholesterol; HDL-C = high density lipoprotein cholesterol; TC = total cholesterol.

Increased values of LDL-C and HDL-C were not significantly related to RHR (Table 3). On the other hand, RHR was positively and significantly related to triglycerides values. In the multivariable model, only triglycerides maintained the significant relationship with RHR, but TC and glucose did not.

Compared to the lowest RHR quartile, the highest quartile was associated with increased values of glucose (OR = 3.82 [OR_95%CI_: 1.11-13.1]; *p* = 0.034) and decreased values of HDL-C (OR = 1.97 [OR_95%CI_: 1.07-3.60]; *p* = 0.028), independent of age, sex and pubertal stage. However, after additional adjustment for cardiorespiratory fitness the multivariable models became non-significant (Table 4).

**Table 4:** Association between resting heart rate and metabolic variables among adolescents (Brazil, n = 971)

	**Binary logistic regression**				
**RHR (beats/min)**	**LDL-C**	**HDL-C**	**Triglycerides**	**TC**	**Glucose**
	**OR (OR**_**95%CI**_**)**	**OR (OR**_**95%CI**_**)**	**OR (OR**_**95%CI**_**)**	**OR (OR**_**95%CI**_**)**	**OR (OR**_**95%CI**_**)**
<70	1.00	1.00	1.00	1.00	1.00
70-77.4	1.24 (0.67-2.28)	1.28 (0.64-2.54)	1.36 (0.12-15.4)	1.21 (0.54-2.71)	1.69 (0.42-6.74)
77.5-85.9	0.99 (0.54-1.79)	1.52 (0.80-2.89)	5.46 (0.68-43.9)	1.01 (0.45-2.22)	2.69 (0.75-9.64)
≥86	1.20 (0.68-2.12)	1.66 (0.88-3.11)	4.04 (0.50-32.3)	1.19 (0.56-2.52)	3.06 (0.88-10.6)

Binary logistic regression adjusted by age, pubertal stage, gender and cardiorespiratory fitness; OR = odds ratio; 95%CI = 95% confidence interval; LDL-C = low density lipoprotein cholesterol; HDL-C = high density lipoprotein cholesterol; TC = total cholesterol; RHR = resting heart rate.

The ROC curve indicated significant potential for the RHR in screening adolescents at an increased value of fasting glucose (AUC = 0.611 ± 0.039) and triglycerides (AUC = 0.618 ± 0.044) (Table 5). On the other hand, the potential for screening decreased values of HDL-C (AUC = 0.518 ± 0.026) and increased values of LDL-C (AUC = 0.525 ± 0.023) and TC (AUC = 0.539 ± 0.028) was limited. RHR was more specific than sensitive for screening the outcomes and the better cutoffs for RHR varied according to the analyzed outcome (except for TC and LDL-C where cutoff = 85.5 beats/min).

**Table 5:** Impact of resting heart rate on screening metabolic variables among adolescents (Brazil, n = 971)

**ROC curve**	**Outcomes**				
	**High LDL-C**	**Low HDL-C**	**High triglycerides**	**High TC**	**High glucose**
AUC ± SE	0.525 ± 0.023	0.518 ± 0.026	0.618 ± 0.044	0.539 ± 0.028	0.611 ± 0.039
AUC_95%CI_	(0.479 – 0.570)	(0.467 – 0.568)	(0.531 – 0.705)	(0.483 – 0.594)	(0.534 – 0.688)
AUC *p*-value	0.293	0.491	0.030	0.195	0.007
Best cutoff (beats/min)	85.5	81.5	84	85.5	89
Sensitivity	0.462	0.563	0.621	0.486	0.491
Specificity	0.620	0.482	0.564	0.615	0.715

ROC = receiver-operating characteristic; AUC = area under ROC curve; SE = standard error; 95%CI = 95% confidence interval; LDL-C = low density lipoprotein cholesterol; HDL-C = high density lipoprotein cholesterol; TC = total cholesterol.

## Discussion

The results of this study indicated that higher RHR was related to lower cardiorespiratory fitness, independent of obesity and other confounders. Moreover, the inclusion of cardiorespiratory fitness as a confounder in logistic regression made the associations of the outcomes with RHR non-significant. Previous studies have reported that the relationship between cardiorespiratory fitness and lipid variables/blood pressure in adolescents is mediated by body fatness, whereas the observed relationships with fatness are independent of cardiorespiratory fitness [20]. Our findings indicate an inverse effect of these confounders on the relationship between RHR and the metabolic outcomes (independent of fatness and strongly dependent on cardiorespiratory fitness).

The close inverse relationship between cardiorespiratory fitness and RHR has been demonstrated in previous reports by other authors [10]. The recognized effect of cardiorespiratory fitness in autonomic nervous system activity and subsequent adaptations in neurohumoral control (decrease in circulating levels of catecholamines and changes in number or affinity of receptors) [21] seems to be independent of body composition [22] and could offer support to our results.

A previous study [22] found that parasympathetic indexes of obese adults engaged in ≥2 hours per week of physical exercises were higher than those observed in sedentary adults of normal weight. Moreover, this protective effect has been identified in children. Gutin et al. [23] identified an improvement in parasympathetic activity in obese children submitted to 8 months of a physical training protocol, which decreased after subsequent detraining (changes in parasympathetic activity were not related to modifications in body fatness). In our study, cardiorespiratory fitness was negatively related to RHR (sport practice only in the univariate model) and, therefore, as previously observed in other cardiovascular and metabolic outcomes [1,2], physical activity practice during early life could be useful in the prevention of excessive weight gain [20], promotion of lower RHR and hence the prevention of cardiovascular diseases in adulthood.

Additionally, high RHR was also associated with sleep pattern. Recently, Gallicchio and Kalesan [24] in a systematic review/meta-analysis identified that people with both short and longer periods of sleep are at an increased risk of all-cause mortality. However, the actual pathway by which sleep is linked to cardiovascular complications [25] is not clear, although it is plausible to believe that a pathway exists. Adolescents are prone to perform more activities at night (TV viewing and computer usage) than children and thus they are more exposed to shorter periods of sleep. Short sleep may act as an acute and chronic stressor and, therefore, affect the sympathetic activity of the organism and lead to an increase in RHR [26]. Moreover, the concentration of pro-inflammatory agents (interleukine-6, tumor necrosis factor – alpha and C-reactive protein) is increased in people with short sleep periods [26]. Our findings highlight the fact that health professionals must target the promotion of adequate sleep patterns among pediatric populations, because this harmful relationship between sleep pattern and a higher RHR seems significant from an early age.

RHR has significant potential for screening increased fasting glucose values. In agreement with this, Dubose et al. [27] recently identified that RHR can be used, together with other variables, to screen American adolescents with glucose impairment. Researches have indicated that insulin resistance has an important relationship with sympathetic activation [28-30], which significantly affects the RHR.

Similarly, dysfunction in lipid metabolism was also related to a high RHR. A previous study [11] found a positive and significant relationship between RHR, triglycerides and TC among obese children and adolescents. On the other hand, the same authors point out that the causality/pathway by which a high RHR is linked to lipid dysfunction cannot be clearly determined. It is plausible to believe that insulin resistance could also be relevant in this process [28]. In fact, insulin resistance affects the process of energy production, leading to an increased use of lipids as fuel and a higher production of reactive oxygen species in the brain (by the activation of the nicotine adenine dinucleotide hydrogen phosphatase oxidase), which increases the oxidative stress in the rostral ventrolateral medulla, the region that determines the basal sympathetic activity [29,30]. Apparently, this inflammatory process occurs irrespective of the presence of obesity and ratifies the potential of RHR for screening adolescents at an increased cardiovascular risk.

Palatini [7] pointed out that among adults there are no doubts that an RHR ≥80 to 85 beats per minutes implies an increased risk for health. In pediatric populations this RHR range seems not to be true, because the cutoffs were different according to outcomes analyzed (ranging from 81.5 to 89). Moreover, previous studies (and also our findings) have reported a significant RHR variation according to age groups [9,11,31]. Therefore, future cutoff tables should be developed in longitudinal observations and take into account adjustments for gender and age.

Our study has strengths, such as the sample size calculation and random process for selecting the schools/classes. However, the limitations must be recognized too. Initially, the absence of dietary habits related to RHR (e.g. cola intake, coffee, energy drinks) constitutes a significant weakness in our study and a target for further investigations. Our sample has a wide age range and the peak height velocity has limitations when applied to some age groups within this range. On the other hand the use of other methods to estimate pubertal stage involves ethical and logistical complications. The absence of measures related to adipokines and insulin resistance must be recognized and should be the focus of future investigations.

Finally, the low magnitude of the correlation coefficient found [32] should be taken into account in further inferences, because it denotes the action of other variables in the relationship between RHR and the analyzed outcomes. Thus, mediated effect could be controlled by the simultaneous use of the RHR together with other variables (e.g. general obesity, abdominal obesity, low cardiorespiratory fitness) to screen adolescents at an increased metabolic risk in further studies [27].

## Conclusions

The present data indicates that a high RHR constitutes a significant and independent risk factor to screen alterations in glucose and triglycerides from early in life, but further studies of RHR cutoffs are necessary. Moreover, cardiorespiratory fitness and sleep were significantly correlated to RHR, independent of a variety of potential confounders.

## Abbreviations

RHR: Resting heart rate; %BF: Body fatness percentage; EDTA: Ethylene-diamine-tetraacetic acid; TC: Total cholesterol; HDL-C: High-density lipoprotein cholesterol; LDL-C: Low-density lipoprotein cholesterol; ROC: Receiver-operating characteristic; AUC: Area under the ROC curve; 95%CI: 95%confidence interval; OR: Odds ratio.

## Competing interests

The authors declare that they have no competing interests.

## Authors’ contributions

RAF and ESC: (1) conception and design of the study, (2) acquisition, analysis and interpretation of data, (3) draft of the article and selection of manuscripts to discuss the results. DV, DRPS, CMTC, MSC and MBB: (1) Acquisition, analysis and interpretation of data, (2) draft of the article and selection of manuscripts to discuss the results, DSB, MJCS, LBS and ERVR: (1) conception and design of the study (2) review and approval of the final version to be submitted. All authors read and approved the final manuscript.

## Pre-publication history

The pre-publication history for this paper can be accessed here:

http://www.biomedcentral.com/1471-2431/13/48/prepub
